# Prognostic Value of CD9 in Solid Tumor: A Systematic Review and Meta-Analysis

**DOI:** 10.3389/fonc.2021.764630

**Published:** 2021-11-19

**Authors:** Ping Zeng, Meng Si, Rui-xia Sun, Xu Cheng, Xiao-yang Li, Min-bin Chen

**Affiliations:** ^1^ Department of Radiation Oncology, the First Affiliated Hospital of the USTC, Division of Life Sciences and Medicine, University of Science and Technology of China, Hefei, China; ^2^ Hefei Cancer Hospital, Chinese Academy of Sciences, Hefei, China; ^3^ Department of Radiotherapy and Oncology, Affiliated Kunshan Hospital of Jiangsu University, Kunshan, China

**Keywords:** CD9, solid tumors, prognosis, overall survival, time to progression

## Abstract

Numerous clinical studies investigated how low expression of CD9 predicts poor prognosis of solid tumor. However, the results were inconclusive. This present meta-analysis was therefore performed to determine the prognostic value of CD9 expression in solid tumors. In this meta-analysis, 25 studies involving 5,555 participants were included; the result showed strong significant associations between declined expression of CD9 and all endpoints: overall survival (OS) (hazard ratio (HR) = 1.88, 95% CI = 1.45–2.43, p < 0.000) and time to progression (TTP) (HR = 2.0, 95% CI = 1.38–2.88, p < 0.000). The subgroup analysis was also performed, which revealed that the associations between CD9 downregulated expression related to poor OS in lung cancer and head and neck cancer. Also, low expression of CD9 was significantly connected with poor TTP in patients with head and neck cancer. The adverse prognostic impact of decreased expression of CD9 was observed in patients of different ethnicities. In conclusion, these results showed that declined expression of CD9 was associated with poor survival in human solid tumors. CD9 may be a valuable prognostic predictive biomarker and a potential therapeutic target in human solid tumors.

## Introduction

CD9, a 24- to 27-kDa cell surface glycoprotein, also known as motility-related protein-1 (MRP-1), leukemia-associated cell surface antigen p24, and TSPAN29, belongs to the transmembrane-4 superfamily (TM4SF), which consists of four transmembrane domains, a small intracellular loop, and two extracellular loops (1, 2). CD9 was initially acknowledged as an antigen for a monoclonal antibody, acquired *via* immunization of mice with pre-B cells, and was subsequently revealed to be ubiquitously expressed on hematopoietic and non-hematopoietic cells (3–5), as well as malignant tumor (1, 2). Through interacting with a variety of cell-surface molecules, CD9 participates in numbers of biological activities including cell adhesion, motility, metastasis (6–8), sperm–egg fusion (9), growth, survival, signal transduction (10, 11), apoptosis (12, 13), and differentiation (14–16).

Many researches indicated that the expression of CD9 was declined in most solid tumors, including lung cancer (17–20), breast carcinoma (21–23), colorectal cancer (24–26), clear cell renal cell carcinoma (27), malignant pleural mesothelioma (28), laryngeal cancer (29), and gastrointestinal stromal tumor (GIST) (30), and that decreased expression of CD9 strongly correlated with the progression, increased risk of recurrence, angiogenesis, and metastasis of some malignant tumors (26, 30, 31), as well as a significantly increased risk of malignancy (32). The study of Lewitowicz has shown that above 98% of GISTs have moderate-to-strong expression of CD9 (33). This proof implied that MRP-1 would be further reckoned as a promising indicator of prognosis of cancer.

A great deal of reports manifested that reducing expressed MRP-1 was associated with dismal prognosis of different cancers. However, in locally advanced gastric cancer, high expression of CD9 was negatively associated with the prognosis (33). The outcome of these individual researches was inconsistent. Consequentially, this meta-analysis was deliberately calculated to illuminate the outcome value of CD9, and this glycoprotein may be a promising therapeutic target in solid tumors.

## Materials and Methods

### Publication Search

This meta-analysis was conducted according to the Preferred Reporting Items for Systematic Reviews and Meta-Analyses guidelines (34). A complete network research was done by electronic databases PubMed, Embase, and Web of Science (up to July 20, 2021) through different combinations of the search items: “CD9,” “MRP1,” and “cancer”/”tumor”/”neoplasm”/”carcinoma” and the following limits: human subjects and reports in the English language. All probably appropriate articles were selected, and their references were prudently browsed to recognize else applicable essay. While several researches of the same patient population existed, we involved the available data with the largest sample size.

### Inclusion Criteria

Reports that meet the following criteria were considered eligible: a) calculated the expression of CD9 for predicting outcome (overall survival (OS) or time to progression (TTP)) in human cancer, b) provided hazard ratios (HRs) with 95% CIs or sufficient information that could estimate it, and c) classified CD9 expression as “high” and “low” or “positive” and “negative.”

### Exclusion Criteria

The literatures were excluded based upon the subsequent standard: a) researches were published as laboratory paper, case reports, letters, editorials, and abstracts, as well as reviews and proficient opinions; b) experiments were done *in vitro* or *in vivo* and not based upon patients; c) literatures without information on HRs, 95% CI of OS and/or disease-free survival (DFS), or even the Kaplan–Meier (K-M) survival curves; d) researches that described the survival outcome of other indicators; and e) unpublished studies.

### Data Extraction

Two reviewers extracted data from the articles independently and carefully using a standardized form. The third author would independently extract data from the original article. If disagreements exist, consensus would be reached through debate. This article was founded on OS and TTP. DFS and time to recurrence (TTR) that had similar definition were merged into TTP of tumor. The following information was extracted from selected papers: the first author, publication year, country of participants, number of patients considered, kinds of cancer, cutoff values, OS, TTP, Newcastle–Ottawa scale (NOS) score. The chief characteristics of these researches are shown in **Table 1**. For some articles, HR can be directly obtained; for the researches in which survival data are presented only with K-M curves, Tierney’s method was used to calculate the HR and 95% CI (46). NOS was employed to evaluate the eligible literature. The scores of eligible articles vary from 6 to 9, which mean that the methodological quality of these papers was high.

**Table 1:** Characteristics of studies included in the meta-analysis.

Author	Year	Country	Case	Disease	Method	Cutoff value	Endpoint	NOS
Kwon HJ (21)	2017	Korea	1,349	Breast carcinoma	IHC	Score > 4	TTP	8
Zheng WQ (17)	2016	China	959	NSCLC	Gene cluster analysis		OS	6
Kim KJ (24)	2016	Korea	304	Colorectal cancer	IHC	Score of 4 to 12	TTP	7
Kwon HJ (27)	2014	Korea	644	Renal carcinoma	IHC	More than 10% positive cells	OS, TTP	7
Amatya VJ (28)	2013	Japan	112	Malignant pleural mesothelioma	IHC	Score ≥ 1+	OS	8
Zhang BH (29)	2013	China	100	Laryngeal carcinomas	IHC	Point ≥ 80	OS	8
Yang HX (30)	2013	China	74	Gastrointestinal stromal tumor	IHC	Score > 3	TTP	8
Zou Q (35)	2012	China	67	Gallbladder cancer	IHC	An average rate of positive cells ≥ 25%	OS	7
Setoguchi T (36)	2011	Japan	104	Gastric GIST	IHC	Scores of 2+ and 3+	TTP	7
Buim MEC (37)	2010	Brazil	179	Oral carcinoma	IHC	Over 10% of the tumor cells were stained;	OS, TTP	8
Soyuer S (38)	2010	Turkey	49	Gastric cancer	IHC	More than 10% of total cancer cells showed cytoplasmic staining	OS, TTP	6
Mhawech P 2 (39)	2004	Switzerland	153	Head and neck carcinoma	IHC	≥50% stained tumor cells	TTP	8
Hashida H (25)	2003	Japan	146	Colon cancer	IHC; RT-PCR	A score of ≥120; the conservation rate value ≥ 0.8	OS, TTP	8
Erovic BM (40)	2003	Austria	34	head and neck carcinoma	RT-PCR, IHC	MRP-1/CD9/HPRT ratio was >3; five hot spots	OS, TTP	8
Mhawech P (41)	2003	Switzerland	229	Bladder carcinoma	IHC	>50% positive cells	TTP	7
Kusukawa J (42)	2001	Japan	78	Oral carcinoma	IHC	The basal cells of the nest were stained	OS	6
Miyamoto S (43)	2001	Japan	56	Endometrial cancers	IHC	Extent of immunoreaction > 50%	TTP	8
Miyake M (18)	1999	Japan	187	NSCLC	RT-PCR	Value > 1.0	OS	8
Shimada Y (44)	1999	Japan	116	Esophageal carcinoma	IHC	More than 50% positive staining in membrane	TTP	7
Sho M (45)	1998	Japan	40	Pancreatic cancer	RT-PCR	Conservation rate value > 1.0	OS	7
Mori M (26)	1998	Japan	82	Colon cancer	RT-PCR	VALUE ≥ 0.5	OS	7
Huang CL (22)	1998	Japan	109	Breast cancer	RT-PCR, IHC	Conservation rate > 1; HScore ≥ 50	OS, TTP	6
Higashiyama M (19)	1997	Japan	132	Lung carcinoma	IHC	The percentage of MRP-1/CD9-positive cancer cells ≥ 50%	OS, TTP	8
Miyake M (23)	1996	Japan	143	Breast carcinoma	WB, IHC	A band intensity > 30%; >50% of the carcinoma cells were positively stained	OS, TTP	8
Higashiyama M (20)	1995	Japan	109	NSCLC	RT-PCR	Value > 0.7	OS	8

NOS, Newcastle–Ottawa scale; IHC, immunohistochemistry; TTP, time to progression; NSCLC, non-small cell lung cancer; OS, overall survival; GIST, gastrointestinal stromal tumor; MRP-1, motility-related protein-1; WB, Western blotting; HPRT, hypoxanthine-guanine phosphoribosyltransferase.

### Statistical Analysis

Stata14.0 (StataCorp, College Station, TX) was used to perform the statistical analysis. Initially, we calculated the connection between MRP-1 and endpoints (OS and TTP) though pooled HRs and 95% CIs. Cochran’s Q test and Higgins I-squared statistic were applied to evaluate whether heterogeneity was present between selected articles. Heterogeneity was considered significant when p < 0.1 or I^2^ > 50% (47), and then a random-effects model was employed to pool the HRs and 95% CIs; otherwise, a fixed-effects model was used (35). Moreover, subgroup analysis was used to explore the source of heterogeneity. Begg’s and Egger’s tests were employed to discover the potential publication bias. When publication bias indeed exists, the Duval and Tweedie trim and fill method (37) was exploited. For the robust estimation of the results of statistical analysis, sensitivity analysis was performed by removing the original study one by one. The p-value for all tests was two-tailed, and p < 0.05 was defined as statistically significant, except for heterogeneity.

## Results

### Demographic Characteristics

**Figure 1:**
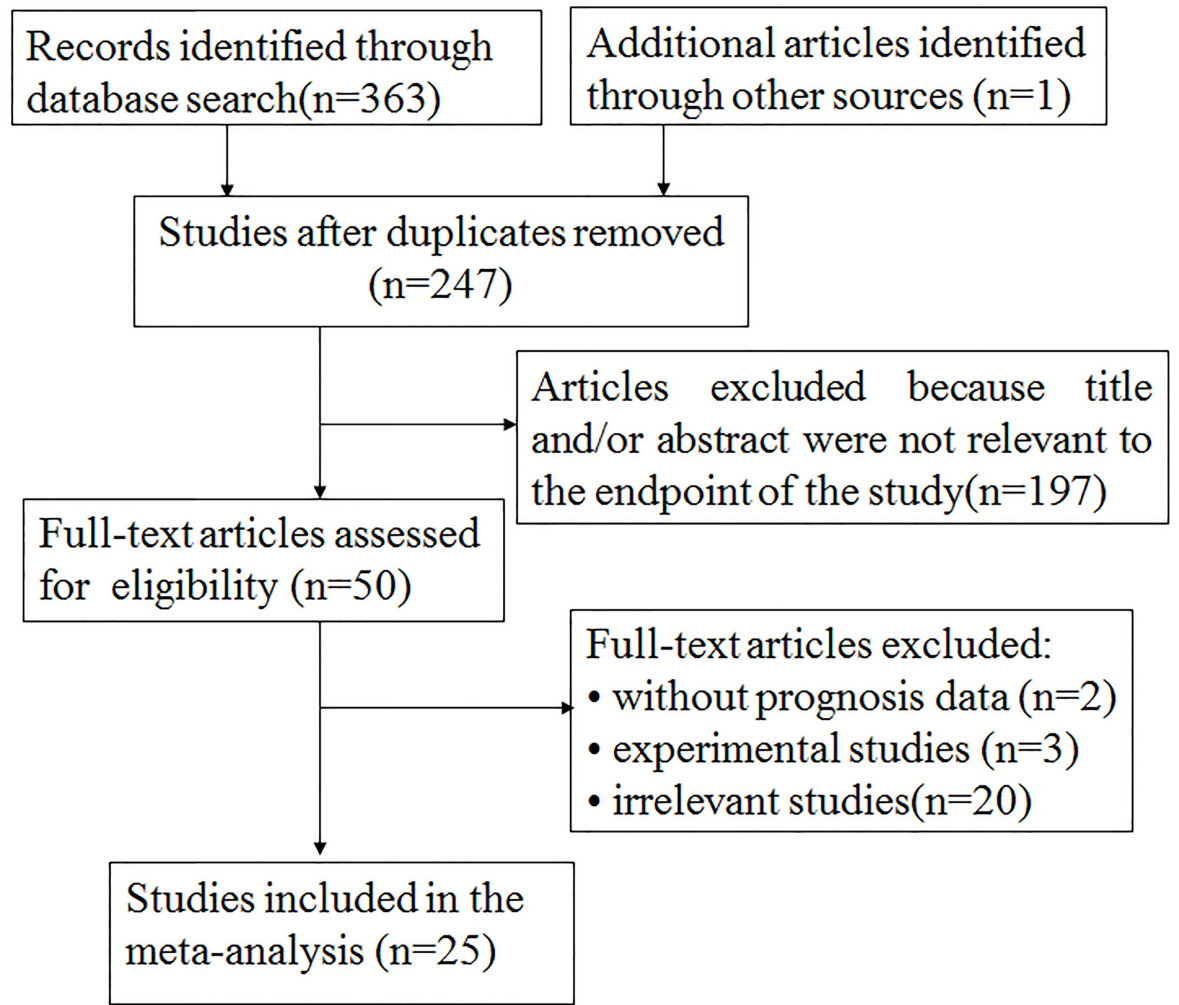
The flowchart of the selection process in our meta-analysis.

The particular process of searching and filtering is displayed in **Figure 1**. We preferentially retrieved 364 records from PubMed, Embase, and Web of Science in accordance with the criteria mentioned previously. Among them, 117 duplicate reports were removed. After the abstracts or full text was scanned, 222 records were excluded by reason of no relevant information provided (n = 197); experimental research (n = 3); without prognosis data (n = 2); and irrelevance (n = 20). Ultimately, 25 records including 5,555 participants were involved in the meta-analysis. The median specimen size was 222, ranging from 34 to 1,349. Among all cohorts, three studies estimated breast carcinoma (21–23), four studies estimated lung cancer (17–20), three studies evaluated colon cancer (24–26), one study estimated clear cell renal cell carcinoma (27), one study estimated malignant pleural mesothelioma (28), one study assessed laryngeal squamous cell carcinomas (29), one study assessed GIST (30), one study evaluated gallbladder cancer (38), one study evaluated gastric GIST (39), two studies investigated oral squamous cell carcinoma (40, 41), one study investigated gastric cancer (42), two studies investigated squamous cell carcinoma of the head and neck (43, 44), one study investigated urothelial carcinoma of the bladder (45), one study investigated endometrial cancers (48), one study investigated esophageal squamous cell carcinoma (49), and one study investigated pancreatic cancer (50). Five studies (25%) were focused on Asians and 20 (75%) on Caucasians. Overall, 17 papers reported on OS and 16 papers on TTP.

### Evidence Synthesis

**Figure 2:**
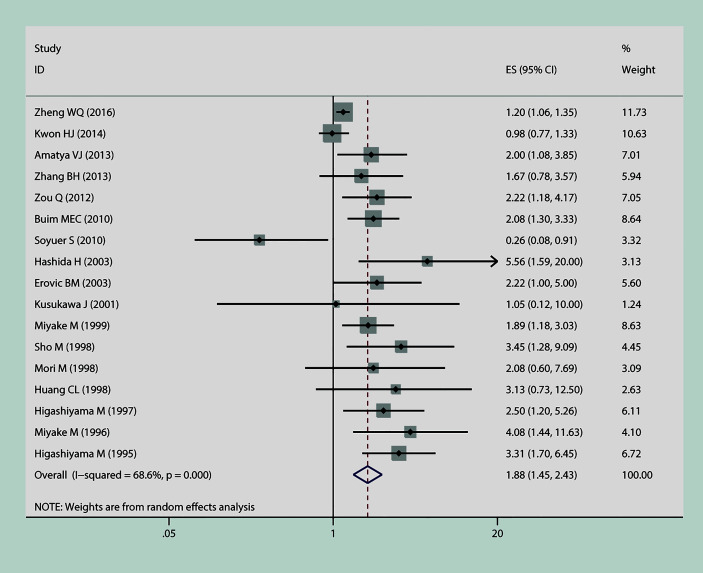
The connection between CD9 expression and overall survival (OS) in solid tumors.

**Figure 3:**
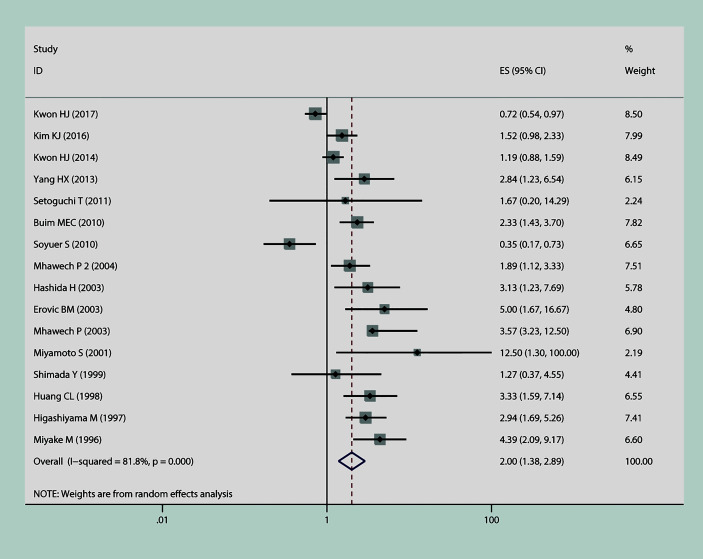
The connection between CD9 expression and time to progression (TTP) in solid tumors.

In this meta-analysis, we estimated the correlation between CD9 and prognosis of tumor. As is shown in **Figure 2**, the result of heterogeneity test illustrated that p < 0.001 and I^2^ = 68.2%; therefore, a random-effects model was applied to evaluate the pooled HR and 95% CI of OS. The outcome implied that declined expression of CD9 was positively correlated with poor OS in solid tumor (pooled HR = 1.88, 95% CI = 1.45–2.43, p = 0.000). As is displayed in **Figure 3**, a random-effects model was employed to calculate the pooled HR and 95% CI of TTP as well; the heterogeneity test reported p < 0.001 and I^2^ = 81.7%. The results indicated that low expression of CD9 was significantly associated with shorter TTP (pooled HR = 2, 95% CI = 1.38–2.88, p = 0.000). To explore the source of heterogeneity, subgroup study was therefore executed. We discovered that decreased expression of CD9 was connected to shorter OS in Asians participants (HR = 1.96, 95% CI = 1.49–2.58, p < 0.001; random effects: I^2^ = 66.5%, p < 0.001), as well as in lung cancer (HR = 1.93, 95% CI = 1.18–3.17, p < 0.001; random effects: I^2^ = 79.3%, p = 0.002) and head and neck cancer (HR = 1.98, 95% CI = 1.39–2.82, p < 0.001; fixed effects: I^2^ = 0%, p = 0.89). The correlation was also detected between decreased expression of CD9 and poor TTP in either Asians patients (HR = 1.38, 95% CI = 1.17–1.62, p < 0.001; random effects: I^2^ = 80.4%, p < 0.001) or Caucasian patients (HR = 1.87, 95% CI = 1.41–2.48, p < 0.001; random effects: I^2^ = 85.4%, p < 0.001), and in patients with head and neck cancer (HR = 2.31, 95% CI = 1.59–3.36, p < 0.001; fixed effects: I^2^ = 11.5%, p = 0.323). No other significant connection between CD9 and the two endpoints was detected in the other subgroup analyses.

### Publication Bias and Sensitivity Analysis

**Figure 4:**
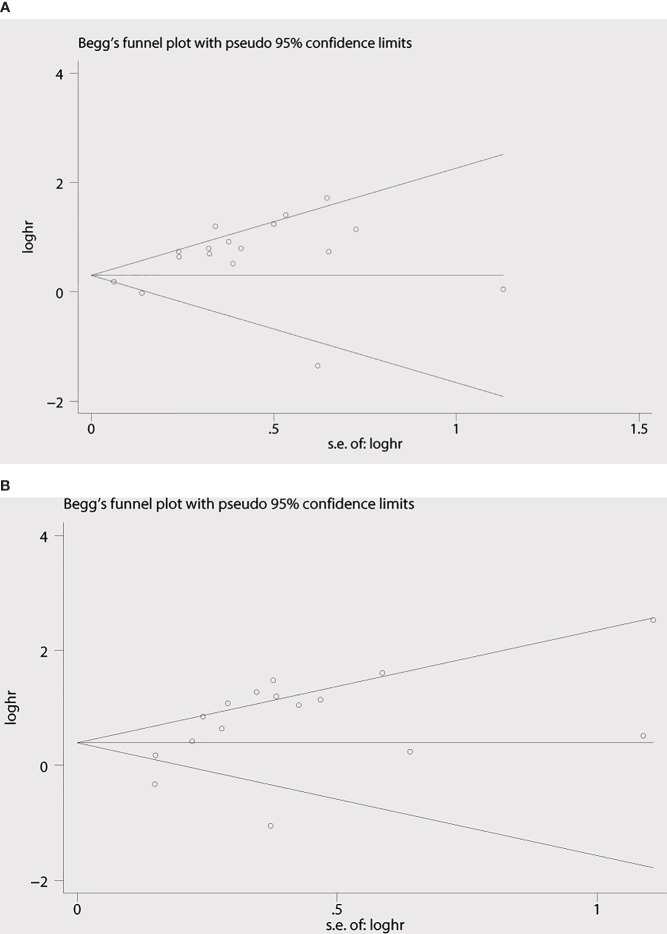
Begg’s funnel plots for the studies involved in the meta-analysis. **(A)** Overall survival. **(B)** Time to progression (TTP). loghr, logarithm of hazard ratios; s.e., standard error.

**Figure 5:**
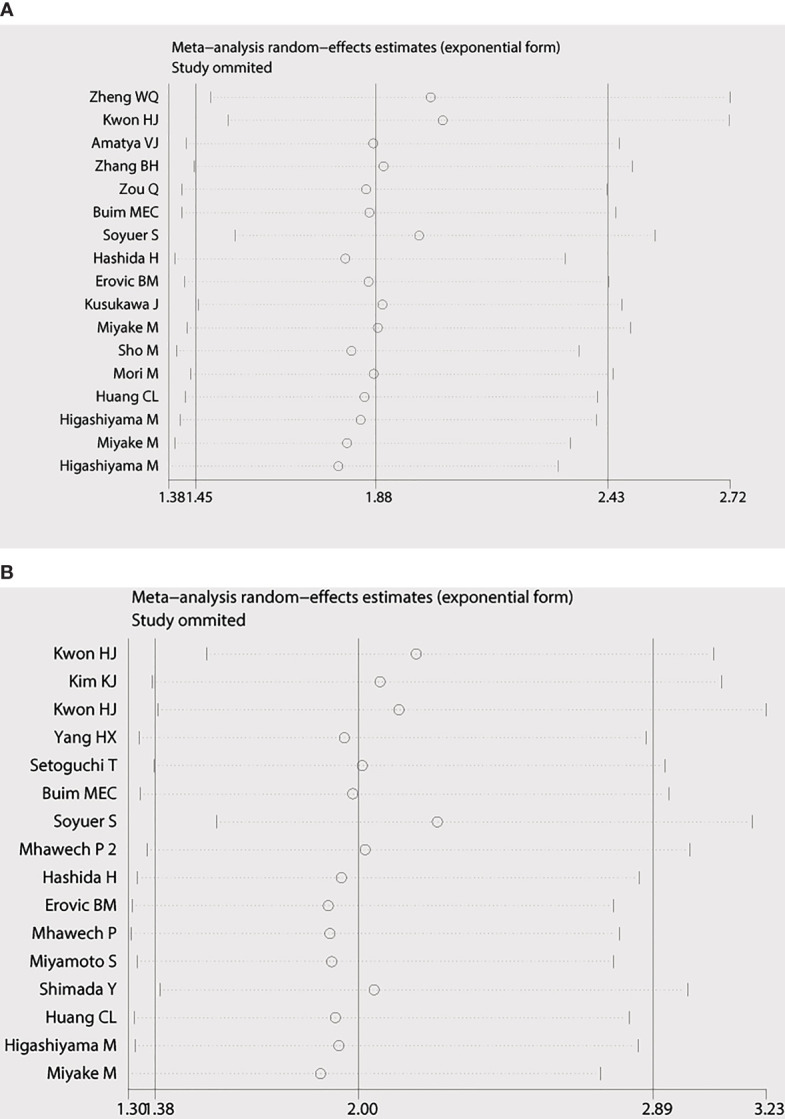
Sensitivity analysis of the meta-analysis. **(A)** Overall survival. **(B)** Time to progression (TTP).

Begg’s funnel plot and Egger’s test were applied to measure the publication bias of the literature. As is shown in **Figure 4A**, Begg’s and Egger’s test scores of OS were correspondingly p = 0.869 and p = 0.008. The funnel plot for the OS showed asymmetry. At the same time, the calculation of the TTP yielded publication biases (Begg’s test, p = 0.86 and Egger’s test, p = 0.024) (**Figure 4B**). Consequently, the trim and fill method was employed to make the pooled HR more dependable, and the result showed that the pooled p-value was also less than 0.01 (figure not shown). Furthermore, sensitivity analysis was performed by removing one study in turn, which revealed that no single study would significantly affect the pooled HRs of OS and TTP (**Figure 5**).

## Discussions

Cancer is the most deadly disease threatening human life. With the increasing incidence of cancer, it is very urgent to explore a therapeutic target. Many clinical studies investigated that declined expression of CD9 was associated with poor prognosis. It is implied that CD9 may be a promising therapeutic target for solid tumors. However, the major individual study had limited participants, and the conclusion was inconsistent. Therefore, we performed this meta-analysis to explore the correlation between CD9 expression and prognosis of solid tumor.

A total of 25 different studies that included 5,555 participants were involved in this current meta-analysis. The results definitely demonstrated that declined expression of MRP-1 was an adverse effect factor to prognosis, with both shorter OS (pooled HR = 1.88, 95% CI = 1.45–2.43, p < 0.001) and TTP (pooled HR = 2.0, 95% CI = 1.38–2.88, p = 0.000). Likewise, the subgroup analysis confirmed that decreased CD9 expression was positively correlated with shorter TTP in Asians and Caucasian patients and shorter OS in Asians patients. When data were sorted in terms of cancer types, the outcome revealed that low expression of CD9 was a poor prognosis indicator for OS in participants with lung cancer and head and neck cancer, as well TTP in patients with head and neck cancer.

This study is so far the first and most comprehensive meta-analysis to systematically explore the possible prognostic role of CD9 downregulation in solid tumors. Our measurable results are intensely in favor of the view that low CD9 expression is associated with poor OS and TTP. In addition, the meta-analysis also reflects the following important implications. First, declined CD9 expression may be a poor outcome indicator in solid tumors. In this article, we involved various cancer types. The aggregate results indicate that reduced CD9 expression is associated with shorter OS and TTP, which can be extended to all solid tumors. Furthermore, it emphasizes that CD9 may be a promising therapeutic target and prognostic indicator for solid tumors.

In addition to the encouraging results, this calculable meta-analysis still has limitations. First, most of the included studies are shown to be retrospective, and positive results are more likely to be published. Moreover, the methods for assessing CD9 expression and cutoff values are inconsistent. Therefore, our results may be overestimated.

Significant heterogeneity also existed in this analysis. Sensitivity and subgroup analyses based on ethnicity and cancer type were conducted to detect the source of the heterogeneities. Heterogeneity was observed in subgroups of race and tumor type included except for head and neck cancer. This suggests that many different factors caused the heterogeneity. First, the method of detection and the year of publication were different. Second, both IHC and semi-quantitative evaluation methods were affected by many factors such as antibody quality, concentration and incubation time, and personal operation methods. Third, the sample source of each study was various. This may be an implicit factor for the existence of heterogeneity.

In conclusion, this meta-analysis clearly demonstrates that decreased CD9 expression in solid tumor tissues is associated with low survival rate. We believe that CD9 may be a helpful prognostic indicator and a promising therapeutic target for solid tumor. However, further studies related to specific tumor types and opinions are needed to confirm the clinical value of CD9 expression in solid tumor.

## Data Availability Statement

The datasets presented in this study can be found in online repositories. The names of the repository/repositories and accession number(s) can be found in the article/supplementary material.

## Author Contributions

M-bC and X-yL designed this study. PZ and MS contributed to the literature search, review, and data extraction. PZ and XC conducted the statistical analyses. PZ, MS, and R-xS contributed to the manuscript drafting. PZ and X-yL contributed to the manuscript revision. All authors contributed to the article and approved the submitted version

## Funding

This work was supported by the Fundamental Research Funds for the Central Universities (WK9110000177); National Natural Science Foundation (81773192, 82072712); Natural Science Foundation of Jiangsu Province (BK20171248); Jiangsu Youth Medical Talents Project (QNRC2016527).

## Conflict of Interest

The authors declare that the research was conducted in the absence of any commercial or financial relationships that could be construed as a potential conflict of interest.

## Publisher’s Note

All claims expressed in this article are solely those of the authors and do not necessarily represent those of their affiliated organizations, or those of the publisher, the editors and the reviewers. Any product that may be evaluated in this article, or claim that may be made by its manufacturer, is not guaranteed or endorsed by the publisher.
